# Early Antioxidative Response to Desiccant-Stimulated Drought Stress in Field-Grown Traditional Wheat Varieties

**DOI:** 10.3390/plants12020249

**Published:** 2023-01-05

**Authors:** Krešimir Dvojković, Ivana Plavšin, Dario Novoselović, Gordana Šimić, Alojzije Lalić, Tihomir Čupić, Daniela Horvat, Marija Viljevac Vuletić

**Affiliations:** 1 Agricultural Institute Osijek, Južno Predgrađe 17, 31000 Osijek, Croatia; 2 Centre of Excellence for Biodiversity and Molecular Plant Breeding (CoE CroP-BioDiv), Svetošimunska Cesta 25, 10000 Zagreb, Croatia

**Keywords:** drought tolerance, wheat, genetic resources, ROS, antioxidative response, phenolics, photosynthetic pigments, antioxidative enzymes

## Abstract

Extended drought affects the production and quality of wheat (*Triticum aestivum* L.), one of the world’s most important food crops. Breeding for increased drought resistance is becoming increasingly important due to the rising demand for food production. Four old traditional Croatian wheat cultivars were used in the present study to examine the early antioxidant response of flag leaves to desiccant-stimulated drought stress and to identify drought-tolerant cultivars accordingly. The results indicate that the enzymatic antioxidant system plays the most significant role in the early response of adult wheat plants to drought stress and the removal of excessive H_2_O_2_, particularly GPOD and APX. Nada and Dubrava cultivars revealed the strongest activation of the enzymatic defense mechanism, which prevented H_2_O_2_ accumulation and lipid peroxidation. Additionally, the Nada cultivar also showed increased synthesis of proline and specific phenolic compounds, which both contribute to the increased stress tolerance. Among the cultivars investigated, cultivar Nada has the broadest genetic base, which may explain why it possesses the ability to activate both enzymatic and non-enzymatic defense mechanisms in an early response to drought stress. This suggests that old traditional wheat cultivars with broad genetic bases can be a valuable source of drought tolerance, which is especially important given the current climate change.

## 1. Introduction

The ongoing climate change is one of the biggest challenges today, threatening global crop production to a greater extent. It has recently been reported that some form of stress (i.e., extreme temperatures, inadequate water availability, and the degradation of chemical, physical, and biological properties of soil) affects a major part of the global cultivation area [1]. Among others, extended drought periods affect the production and quality of wheat (*Triticum aestivum* L.), one of the most important food crops, which accounts for roughly 20% of the caloric intake of the world’s population [2,3]. Within this context, the Croatian region is no exception. Significant yield loss has been reported for Croatian wheat cultivars in recent years due to the more frequent occurrence of prolonged dry periods [4,5]. Therefore, with the increasing need for food production, breeding for improved drought tolerance is becoming of the utmost importance. In the light of global climate changes, cultivars with increased drought tolerance will be of interest as a foundation for the development of future tolerant varieties. Drought tolerant plant varieties are usually characterized by higher levels of osmoprotectants and possess the ability for the prompt and efficient activation of antioxidant machinery [6]. Increased antioxidant activity helps to avoid or reduce oxidative damage caused by an excessive production of reactive oxygen species (ROS) following exposure to drought stress. According to some research, a direct association exists between the plant cultivar’s level of drought tolerance and the degree of antioxidant machinery activation [7]. Traditional cultivars and old germplasm usually harness broader genetic variability and may provide an excellent genetic source of enhanced drought resistance [8,9]. Hence, it is essential to gain insight into the stress response mechanism of these cultivars in order to be able to determine those possessing enhanced antioxidant levels and potentially increased drought tolerance.

Exposure to different biotic and abiotic stress factors such as pathogens, water deficit, salinity, heavy metals, pesticides, extreme temperatures, etc., often leads to an imbalance between the production and scavenging of ROS. Although important, due to their role as signaling molecules in response to various abiotic stresses, the presence of higher concentrations of ROS in plant cells may cause structural and functional damage to important biomolecules, consequently leading to cell and plant death [10]. One of the most harmful processes initiated by the attack of free radicals is lipid peroxidation. It occurs as a result of disturbed ROS balance, disrupting normal cellular function while further exacerbating oxidative stress by generating lipid-derived radicals [11,12]. Lipid peroxidation causes the degradation of both cellular and organelle membranes, compromising their integrity and selectivity. Products of lipid peroxidation are used as markers to assess the severity of lipid peroxidation, among which malondialdehyde (MDA) is the most extensively explored in plant cells [13]. One of the essential characteristics of drought tolerant wheat cultivars is the potential to avoid membrane integrity weakening and mitigate lipid peroxidation following exposure to drought [14], indicated by a lower level of MDA [15]. Exposure to osmotic stress conditions, such as salinity and drought, can also affect leaf protein concentrations [16]. Increased total protein content may indicate a stronger activation of the enzymatic antioxidant system, accumulation of cell proteins, and/or induced synthesis of drought-responsive proteins [17].

To cope with an excessive production of ROS and prevent cell damage, plants have developed defense systems including various enzymatic and non-enzymatic antioxidants [18]. The antioxidant system has a role in quenching excess ROS and providing protection against oxidative stress. Even though it is not the most reactive, H_2_O_2_ has one of the longest half-lives of all ROS [11]. It is widely known that an excess of H_2_O_2_ in plant cells promotes oxidative stress due to the generation of highly reactive hydroxyl radicals [19]. In plant cells, H_2_O_2_ has two contrasting purposes. It is a key signaling molecule involved in the development of resistance to abiotic stressors, but it can also trigger programmed cell death when present in higher concentrations [20]. Among the numerous enzymatic antioxidants, catalase (CAT), ascorbate peroxidase (APX), and guaiacol peroxidase (GPOD) are the ones involved in the regulation of intracellular levels of H_2_O_2_, although using different mechanisms [11]. CAT has the potential to directly dissociate H_2_O_2_ into H_2_O and O_2_, while APX and GPOD use low molecular weight antioxidants as electron donors. Particularly, GPOD can utilize both glutathione (GSH) and thioredoxin as reducing substrates, while APX utilizes ascorbate (ASH) and has a role in scavenging H_2_O_2_ in water–water and ASH-GSH cycles [21,22,23]. Even though CAT has a higher turnover rate, APX has a greater affinity for H_2_O_2_ and seems to be of greater importance in regulating ROS under stress conditions [11]. The ability of plants to tackle the overproduction of ROS and develop tolerance to drought stress has been highly associated with the increased activity of CAT, APX, and GPOD [14,24].

Non-enzymatic antioxidants are compounds that can quickly inactivate free radicals by interrupting the chain reaction they’re involved in. The plant non-enzymatic antioxidant system comprises compounds such as ASH, GSH, phenolic compounds, carotenoids (Car), tocopherols, etc. Phenolic compounds are a highly heterogeneous group of plant secondary metabolites with a high capacity to capture free radicals due to their specific structure. Among other functions, they possess the ability to directly trap ^1^O_2_ and lipid alkoxy radicals, hence preventing the lipid peroxidation cascade [22]. Because of their multifarious roles in ROS scavenging, it has been proven that enhanced accumulation of phenolic compounds under various abiotic and biotic stress conditions significantly contributes to the antioxidant capacity of plants [16,25]. Wheat cultivars with an elevated phenolic content seem to be generally more tolerant to drought conditions [17,26,27]. Polyphenol oxidase (PPO) is one of the enzymes involved in the metabolism of phenolic compounds by oxidizing various phenolic compounds to quinones [28]. Although PPO activity is mostly associated with a plant defense mechanism against biotic stress, several lines of evidence suggest its role in response to abiotic stress as well. It has been demonstrated that extreme growth conditions, such as high temperatures or drought, inhibit the oxidation of phenolic compounds, which leads to an increase in their accumulation while simultaneously resulting in a decrease in PPO activity [29,30]. The exact role of PPO in response to abiotic stress is, however, still largely unknown, as some findings suggest that drought stress causes a significant increase in PPO activity [31].

The mechanism of osmotic adjustment is one of the key physiological mechanisms that plants have developed to cope with osmotic stress, such as increased salinity and drought [32]. Under stress conditions, the accumulation of various osmolytes helps to maintain the cell’s physiological functions. One of the key osmolytes playing a significant role in the tolerance of plants to drought conditions is proline. Besides its role as an osmoprotectant, proline has been shown to be involved in lipid peroxidation inhibition, ROS scavenging, and the prevention of cell death [33,34]. Elevated amounts of proline in wheat have been associated with enhanced drought tolerance, and it has been proven that proline concentration can be an efficient tool for selection of drought tolerant wheat genotypes [35].

The majority of the research examines the tolerance of seedlings to drought stress, which is less relevant in practical crop breeding. Due to strong recovery mechanisms present in young plants, genotypes with lower tolerance to abiotic stress estimated under laboratory or growth chamber conditions at the seedling stage may still exhibit good productivity in the field and vice versa [36]. For example, a study by Dodig et al. [37] revealed low correlations between adult traits (e.g., grain yield, hectoliter weight, thousand grain weight) under pre- and post-anthesis water stress, and seedling traits (e.g., germination percentage and time, coleoptile, and shoot length) under laboratory water stress conditions. For the purpose of wheat breeding for improved drought tolerance, determining the level of tolerance of cultivars in the adult stage, and notably the resistance at critical growth stages that determine productivity of a cultivar, such as the grain filling stage, is of greater importance. The aim of the present study was to examine the early antioxidant response of adult wheat plants (at the level of flag leaf response) to desiccant-stimulated drought stress and to detect cultivars with greater tolerance to drought accordingly. In this study, four old Croatian wheat cultivars were used to determine the potential of old traditional cultivars as a source of enhanced drought tolerance.

## 2. Results

### 2.1. Determination of Stress Occurrence and Severity

**Figure 1:**
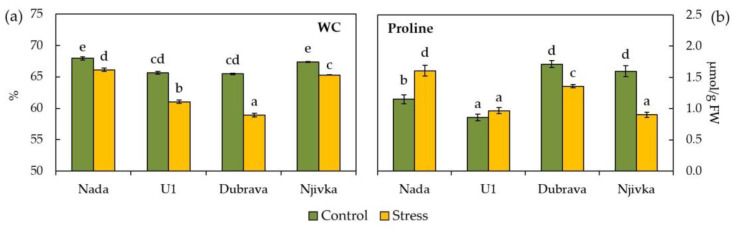
Water content (WC) (**a**) and proline (**b**) in control and drought stressed flag leaves of wheat cultivars. Bars represent mean values ± SE. Different letters indicate significant difference among treatments and cultivars at *p* < 0.05 according to LSD test.

Reliable indicators of drought stress in plant leaves, namely water content (WC) and proline accumulation, were evaluated to identify stress occurrence and severity in wheat flag leaves (Figure 1). WC significantly decreased in leaf tissue of all cultivars collected four hours after stress imposition (Figure 1a) compared to control ones, the least in cultivar Nada (2.72%), and the most in cultivar Dubrava (10.03%). Furthermore, flag leaves of control plants of cultivars Nada and Njivka had the same WC (67.96 and 67.37%, respectively), as did cultivars U1 and Dubrava (65.66 and 65.46%, respectively).

Proline accumulation (Figure 1b) was significantly enhanced in drought stressed flag leaves only in cultivar Nada (39.64% compared to control), while in cultivars Dubrava and Njivka it was significantly lowered (20.62 and 43.67%, respectively) compared to control flag leaves of the same cultivar. In the flag leaves of cultivar U1, no significant change in proline accumulation was found between drought stressed and control ones. Among investigated cultivars, the accumulation of free proline in control flag leaves was the lowest in cultivar U1 (0.86 μmol/g FW) and the highest in cultivars Dubrava and Njivka (1.71 and 1.60 μmol/g FW, respectively).

### 2.2. Hydrogen Peroxide, Lipid Peroxidation and Protein Content

**Figure 2:**
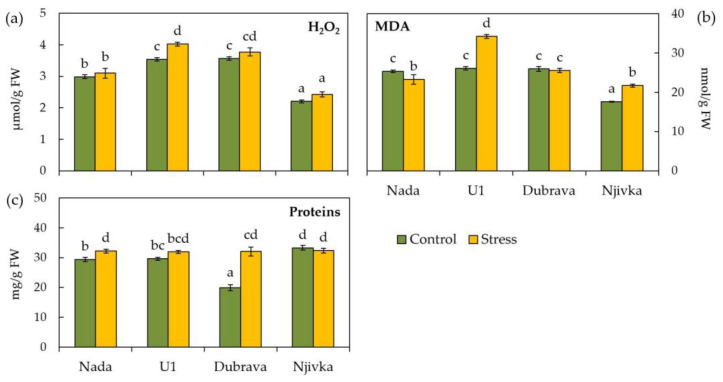
Hydrogen peroxide (**a**), malondialdehyde (MDA) (**b**), and proteins content (**c**) in control and drought stressed flag leaves of wheat cultivars. Bars represent mean values ± SE. Different letters indicate significant difference among treatments and cultivars at *p* < 0.05 according to LSD test.

Drought stress induced a significant accumulation of H_2_O_2_ only in the flag leaves of cultivar U1 (Figure 2a) compared to the control (13.62%). In the drought stressed flag leaves of other investigated cultivars, no significant change in H_2_O_2_ content was found compared to the control. Among investigated cultivars, H_2_O_2_ content in control flag leaves was the lowest in cultivar Njivka (2.20 μmol/g FW) and the highest in cultivars U1 and Dubrava (3.54 and 3.57 μmol/g FW, respectively).

Lipid peroxidation, measured as MDA content, significantly decreased in drought-stressed leaves of cultivar Nada (Figure 2b), while in stressed leaves of cultivars U1 and Njivka, MDA content was significantly increased compared to control and amounted to 31.21 and 23.54%, respectively. Only in the flag leaves of cultivar Dubrava did the MDA content remain at the same level despite the drought stress exposure. MDA content in control flag leaves of cultivars Nada, U1, and Dubrava was almost equal (25.33, 26.07 and 25.93 nmol/g FW, respectively), while in cultivar Njivka it was significantly lower (17.57 nmol/g FW).

The concentration of total soluble proteins in flag leaves of wheat cultivars (Figure 2c) was affected by drought stress only in cultivars Nada and Dubrava, which responded to stress by enhancing the protein content (9.39 and 61.39%, respectively) compared to the control. Moreover, the concentration of soluble proteins in control flag leaves was the lowest in cultivar Dubrava (19.87 mg/g FW) and the highest in cultivar Njivka (33.29 mg/g FW).

### 2.3. Enzymatic Activity

**Figure 3:**
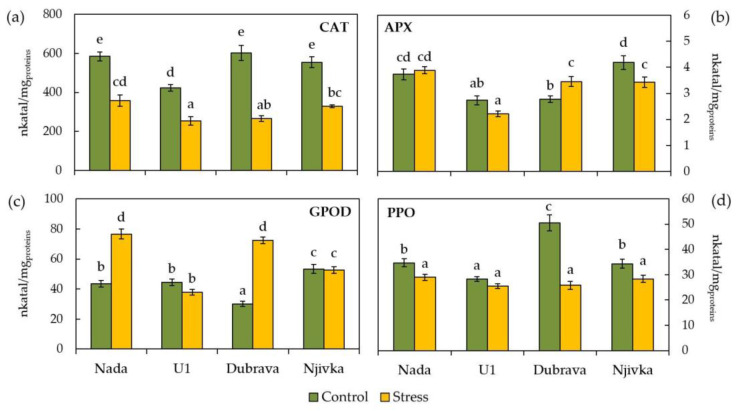
Activities of catalase (CAT) (**a**), ascorbat peroxidase (APX) (**b**), guaiacol peroxidase (GPOD) (**c**), and polyphenol oxidase (PPO) (**d**) in control and drought-stressed flag leaves of wheat cultivars. Bars represent mean values ± SE. Different letters indicate a significant difference among treatments and cultivars at *p* < 0.05 according to LSD test.

Changes in enzymatic activity caused by the exposure of wheat flag leaves to drought stress were detected by analyzing the activities of antioxidative enzymes (CAT, APX, GPOD) and PPO (Figure 3). CAT activity significantly decreased in drought-stressed leaves of all cultivars (Figure 3a) compared to the control ones, the least in cultivar Nada (38.87%) and the most in cultivar Dubrava (55.95%). Furthermore, cultivars Nada, Dubrava, and Njivka had the same level of CAT activity in control leaves (584.82, 602.49, and 554.52 nkatal/mg_proteins_, respectively), while cultivar U1 had lower CAT activity (422.79 nkatal/mg_proteins_).

The antioxidant enzyme APX responded to drought stress only in flag leaves of cultivars Dubrava and Njivka (Figure 3b), in such a way that APX activity in stressed leaves of cultivar Dubrava increased (24.51%), while in Njivka it decreased (18.06%) compared to the control. In addition, cultivars U1 and Dubrava had the same level of APX activity in control leaves (2.74 and 2.77 nkatal/mg_proteins_, respectively), but were lower than cultivars Nada and Njivka (3.73 and 4.18 nkatal/mg_proteins_, respectively), which were also at the same significance level.

GPOD activity in flag leaves of wheat cultivars (Figure 3c) was affected by drought stress only in cultivars Nada and Dubrava, which responded to stress by reinforcing GPOD activity compared to the control for a significant 75.41 and 141.04%, respectively. Moreover, GPOD activity in control flag leaves was the lowest in cultivar Dubrava (30.10 nkatal/mg_proteins_) and the highest in cultivar Njivka (53.33 nkatal/mg_proteins_). Control flag leaves of cultivars Nada and U1 had the same significance level of GPOD activity.

PPO, as an enzyme involved in polyphenolic metabolism, reacted to drought stress conditions in all cultivars except cultivar U1 (Figure 3d). Significant diminution of PPO activity was most prominent in stressed flag leaves of cultivar Dubrava (48.84%), followed by Njivka (17.23%) and Nada (16.53%). In the control wheat flag leaves, PPO activity was at the same significance level in cultivars Nada and Njivka (34.70 and 34.27 nkatal/mg_proteins_, respectively), while cultivars U1 and Dubrava had the lowest (28.34 nkatal/mg_proteins_) and the highest PPO activity (50.46 nkatal/mg_proteins_), respectively.

### 2.4. Total Phenolics and Phenolic Acids Content

**Figure 4:**
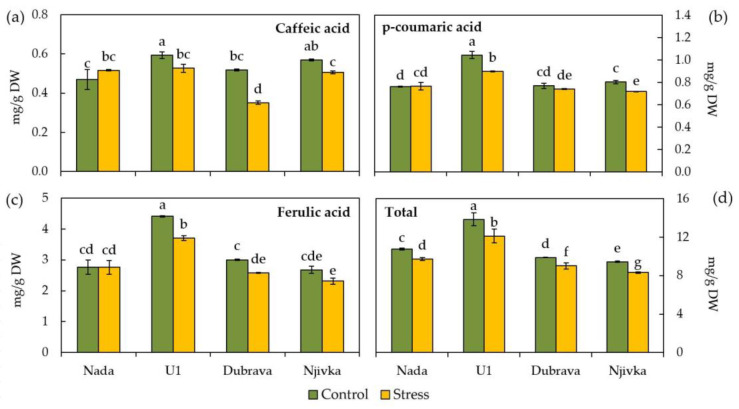
Caffeic acid (**a**), p-coumaric acid (**b**), ferulic acid (**c**), and total phenolic content (**d**) in control and drought stressed flag leaves of wheat cultivars. Bars represent mean values ± SE. Different letters indicate a significant difference among treatments and cultivars at *p* < 0.05 according to LSD test.

The response of total and individual phenolic acids, namely caffeic, p-coumaric, and ferulic acids, to drought stress conditions compared to control ones in wheat flag leaves is shown in Figure 4. In general, control flag leaves of cultivar U1 had the highest content of total and all individual phenolic acids compared to the flag leaves of other three cultivars. Although higher in U1, the difference in caffeic acid content between U1 and Njivka flag leaves was not significant.

All investigated cultivars had different levels of total phenolic content in control flag leaves, as follows: the lowest was found in control leaves of cultivar Njivka (9.44 mg/g DW), while the highest was found in flag leaves of cultivar U1 (13.84 mg/g DW). Drought stress caused a significant decrease in total phenolic content in flag leaves of all four investigated cultivars (Figure 4d), which ranged between 8.58 and 12.46% in the case of Nada and U1 cultivars, respectively.

The impact of drought stress on individual phenolic compounds was not as consistent as in the case of total phenolic content. Caffeic acid content responded to drought stress in flag leaves of all cultivars (Figure 4a), in a way that its content in stressed leaves of cultivar Nada increased (by 9.96%, yet not significantly) while in the remaining three cultivars it significantly decreased (10.90–32.0%) compared to the control, being the most severe in Dubrava cultivar. A similar trend was observed in the case of p-coumaric acid, although at a lower magnitude (Figure 4b). As a result of drought stress, p-coumaric acid content in flag leaves increased by only 0.59% in Nada cultivar and decreased by 3.56, 10.43, and 14.01% in Dubrava, Njivka, and U1 cultivars, respectively. Ferulic acid content in stressed leaves of all cultivars decreased in general compared to the control, even though it was not significant in the case of Nada and Njivka cultivars (0.07 and 13.77% decrease, respectively). The most severe, significant decrease of ferulic acid content was in the case of cultivar U1 (15.97%), followed by cultivar Dubrava (14.04%). Among inspected individual phenolic acids, ferulic acid was the most abundant one, contributing to the total phenolic content in the range between 25.71 and 31.86% in control conditions, and in the range of 27.79 to 30.59% in drought stress conditions.

### 2.5. Photosynthetic Pigments

**Figure 5:**
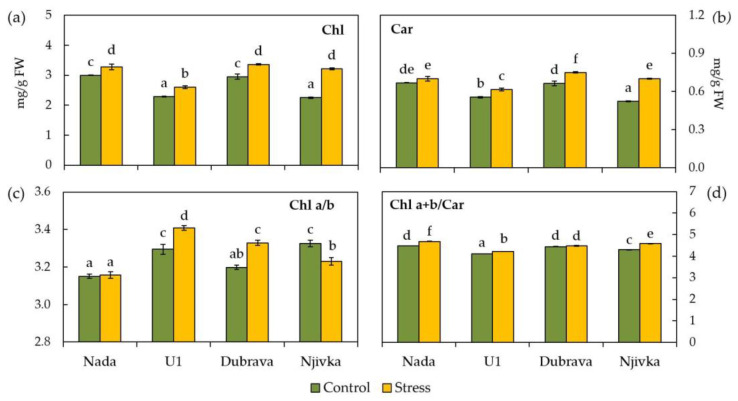
Total chlorophylls (Chl *a*+*b*) (**a**), carotenoids (Car) (**b**), chlorophyll *a*/*b* ratio (Chl *a*/*b*) (**c**), and total chlorophylls/carotenoids (**d**) in control and drought stressed flag leaves of wheat cultivars. Bars represent mean values ± SE. Different letters indicate a significant difference among treatments and cultivars at *p* < 0.05 according to LSD test.

The response of photosynthetic pigments to drought stress in wheat flag leaves is shown in Figure 5. Total chlorophyll content (Chl *a*+*b*) significantly increased in the drought-stressed flag leaves of all cultivars (Figure 5a) compared to control ones, the least in cultivar Nada (9.28%) and the most in cultivar Njivka (42.89%). Furthermore, cultivars Nada and Dubrava had the same significance level of Chl *a*+*b* in control flag leaves (3.00 and 2.95 mg/g FW, respectively) as well as cultivars U1 and Njivka (2.29 and 2.25 mg/g FW, respectively).

The concentration of Car did not respond to drought stress in the flag leaves of cultivar Nada compared to the control, while the other three investigated cultivars in the flag leaves of stressed plants had increased Car content compared to control ones (Figure 5b). The smallest increment in Car content was found in drought-stressed flag leaves of cultivar U1 (10.77%), while the largest one was found in flag leaves of cultivar Njivka (33.93%). In the control flag leaves of cultivar Njivka, the concentration of Car was the lowest (0.52 mg/g FW), while in the control leaves of cultivars Nada and Dubrava were the highest (0.67 and 0.66 mg/g FW, respectively).

Significant differences in Chl *a*/*b* in stressed flag leaves compared to control leaves were found in cultivars U1, Dubrava, and Njivka (Figure 5c). Under drought stress conditions, Chl *a*/*b* in the flag leaves of cultivars U1 and Dubrava increased by 3.41 and 4.08%, respectively, while in cultivar Njivka it decreased by 2.84% compared to control. Moreover, the smallest Chl *a*/*b* in control flag leaves was determined in the case of cultivars Nada and Dubrava (3.15 and 3.20, respectively), while the greatest Chl *a*/*b* was found in flag leaves of cultivars U1 and Njivka (3.30 and 3.32, respectively).

The Chl *a*+*b*/Car ratio in control flag leaves of all four cultivars was almost equal and ranged between 4.11, in the case of U1 cultivar, and 4.48 in the case of Nada cultivar. Although significant for all cultivars except Dubrava, the increase in the Chl *a*+*b*/Car ratio under drought stress conditions was not severe, and it amounted to a maximum of 6.74% in the case of the cultivar Njivka.

## 3. Discussion

In most regions of the world, extensive droughts triggered by climate change are predicted to intensify within the next few decades [9]. An increase in length of duration of dry periods in Eastern Europe and indications for prolonged dry periods in continental Croatia were reported recently by Breinl et al. [38]. Therefore, improvement in drought tolerance in wheat will be of great importance in the following years. Once identified, drought-tolerant traditional wheat cultivars may serve as a valuable genetic resource to be utilized in breeding programs. Here, we examined the antioxidative response of four traditional winter wheat cultivars by exposing them to desiccant to artificially provoke drought stress conditions in flag leaves and identify those with potentially higher tolerance. We were particularly interested in the response of wheat flag leaf to drought since it has a crucial role in providing the assimilates for grain filling and determining grain yield [39]. The cultivars selected for this study were used in the commercial wheat production in Croatia in the period from 1936 to the end of 1990s, and they differ in respect of genetic background (Figures S1–S4 in the Supplementary Material). The experiment was conducted at the grain filling stage since it is widely known that wheat is particularly sensitive to drought during anthesis and grain filling stages [40,41].

The leaf WC is one of the first signs of water availability and stress occurrence in plants [42,43]. According to some previous studies on wheat, it can be successfully used as a tool for screening and the selection of drought-tolerant cultivars [44,45]. In the present study, flag leaves of all four cultivars responded to simulated drought conditions in a significant decrease of WC compared to the control. A decrease in WC, i.e., an increase of dry matter content, indicates the activation of stress defense mechanisms. Since all cultivars responded in a similar way, the WC content itself could not be used to fully distinguish potentially tolerant from susceptible cultivars in this case. The most severe response was in the case of cultivar Dubrava, while cultivar Nada showed only a slight decrease in WC under stress conditions. As reported by Larbi and Mekliche [46], wheat varieties tolerant to water deficit during the grain filling period exhibit a less extreme water loss, in other word, have higher WC compared to susceptible varieties. This may be due to the higher accumulation of osmoprotectants and osmotic adjustment. Accumulation of an osmoprotectant proline in wheat contributes to an increased osmotic stress tolerance and, hence, helps to screen for drought-tolerant wheat genotypes [47]. According to the results of Bowne et al. [48], proline concentrations varied substantially in flag leaves among different wheat cultivars, but a significant increase compared to the control occurred in all drought-tolerant cultivars. In the present study, only flag leaves of cultivar Nada showed a significant increase in proline concentration under stress conditions, while in cultivar U1 no significant change was observed. This is in accordance with a lower decrease of WC observed in cultivar Nada. Therefore, a significant accumulation of proline in Nada cultivar shortly after the stress occurred may be the first sign of exhibiting a good drought tolerance [49,50]. On the other hand, cultivar Dubrava, and especially cultivar Njivka, did not activate the defense mechanism that includes proline biosynthesis in such short period which consequently resulted in a significant decrease in its concentration. Such a response suggests that these two cultivars do not respond adequately to osmotic stress, hence they may possess a higher susceptibility to drought stress.

Taking into consideration H_2_O_2_, MDA, and protein concentrations, the investigated cultivars can be classified into two groups regarding the similarity in response of flag leaves to drought stress. Namely, cultivars Nada and Dubrava exhibited a significant decrease or no change in MDA concentrations, respectively, concomitant with a significant increase in protein concentration (Figure 2b,c). On the contrary, drought stress caused a significant increase in MDA concentrations in the flag leaves of cultivars U1 and Njivka accompanied with no change in protein concentration. The highest increase in MDA concentration was recorded for cultivar U1, which may be a consequence of the significant increase in H_2_O_2_ concentration due to stress conditions that were observed only in the case of U1. Since they are considered one of the first targets of ROS, a reduction in protein content often occurs as a result of exposure to osmotic stress [16]. Three of the four cultivars used in this research showed negligible changes in protein concentration in response to stress conditions. On the contrary, drought treatment led to a significant increase (1.64 fold) in the protein content of the cultivar Dubrava. The same genotype also exhibited the greatest decrease (1.12 fold) in flag leaf water content, i.e., an increase in dry matter, after treatment as compared to the control. A significant increment in dry matter together with elevated total protein content implies the possibility of enhanced protein synthesis, although an increase in protein content after drought exposure is not a common response. Additionally, an increase in protein concentration may be a sign of stronger activation of an enzymatic defense mechanism against ROS [17]. However, additional research, including proteomic analysis, is required to determine in greater detail, the sources of increased protein content of the Dubrava cultivar following drought exposure and to obtain more conclusive results. Increased MDA content is one of the main signs of an ongoing lipid peroxidation, which leads to the loss of cell membrane integrity due to compositional changes of proteins and lipids [11]. The occurrence of lipid peroxidation is considered an important indicator of oxidative stress in plants that may serve as a biomarker of susceptibility to various stress conditions [51]. According to some previous studies, susceptible wheat varieties exhibited higher levels of peroxidation compared to tolerant ones when exposed to drought stress [15,26,52,53]. Therefore, cultivars Nada and Dubrava may be classified as drought-tolerant to some extent, while cultivars U1 and Njivka tend to be susceptible to drought. These assumptions are in accordance with the recent findings of Peršić et al. [54] who investigated Croatian wheat genotypes and found that MDA level indicated drought tolerance, or precisely, the ability of genotypes to acclimate and overcome the negative impact of drought.

The antioxidant machinery of plants involves numerous enzymatic and non-enzymatic components that reduce potential harmful effects of ROS overproduction under stress conditions in plant cells [16,55,56]. In the present study involvement of CAT, APX, GPOD, and PPO were investigated in an early response of wheat flag leaves to induced drought stress. Although being important in reducing H_2_O_2_ levels under various stress conditions in plants, including wheat [14], the activity of CAT was suppressed under drought stress conditions compared to the control in all cultivars investigated within this study. Similar results were obtained by Naderi et. al. [17] in the case of the exposure of wheat cultivars to severe drought stress. Decreased CAT activity in some Croatian wheat cultivars subjected to drought stress was also observed recently by Vuković et al. [57]. Although the aforementioned studies demonstrated a similar response of CAT activity in wheat subjected to drought stress as the present investigation, there are a few important methodological distinctions to keep in mind. Namely, the studies cited earlier were conducted under growth chamber conditions while this study presents the results obtained under field growing conditions. Furthermore, the study by Vuković et al. [57] examined the response of wheat seedlings to drought stress, while the main focus of the present study was set to the response of flag leaf as one of the main photosynthesis sites in wheat determining the grain filling rate and yield [39]. On the contrary to CAT, APX and especially GPOD showed a higher sensitivity to drought stress, which is not surprising taking into account the higher affinity of APX for H_2_O_2_ compared to CAT [11]. A stronger response of GPOD to drought stress compared to APX in wheat was also reported by Pour-Benab et al. [58] at the seedling growth stage. The activities of APX and GPOD were significantly higher in cultivars Nada and Dubrava in stress conditions compared to control ones, indicating that these two enzymes played a significant role in maintaining the H_2_O_2_ level stable and prevention of lipid peroxidation, and thus improved drought tolerance. Additionally, no response or decrease of APX and GPOD activity under drought stress observed in cultivars U1 and Njivka resulted in an increase in H_2_O_2_ concentration and thus in the MDA level, i.e., the occurrence of lipid peroxidation. These findings suggest that GPOD, together with APX, has a key function during the initial response of wheat flag leaves to drought stress.

Although the exact physiological function of PPO in normal plant development is still mostly unknown, it is believed to be involved in the biosynthesis of pigments [59,60]. Some evidence exists to support the involvement of PPO in the formation of dark melanin pigment [28]. On the other hand, it is well known that PPO catalyzes the oxidation of phenolic compounds into quinones, which are responsible for physiological browning in plants [61]. Among four cultivars investigated within this study, a significantly elevated level of PPO activity in flag leaves of control plants was observed in the case of the Dubrava cultivar, which exhibited 1.45- to 1.78-times higher PPO activity compared to other cultivars. One of the possible explanations is the slight brown-red pigmentation of the stem and flag leaf tips that was observed to naturally occur in the Dubrava cultivar during development, which was not the case in the other three cultivars. However, this phenomenon requires further investigation to gain a clearer insight into the potential causes of such pigmentation in the Dubrava cultivar in order to be able to make more certain conclusions about the potential role of PPO in it. Regarding the influence of changing PPO activity on tolerance to various abiotic stressors, published research findings are still mainly in dispute. Some evidence points to a possible role for PPO in the protection of the photosynthetic apparatus in plants subjected to abiotic stressors, but the exact mechanism has not yet been clarified [62]. Results presented within this study showed that desiccant-stimulated drought conditions decreased PPO activity in flag leaves of each of the four cultivars investigated.

Since PPO is involved in the oxidation of phenolics, assumptions have been made that a substantial decrease in its activity would prevent the oxidation of phenolic compounds and increase their content in plant cells [30]. However, in the case of the cultivars examined in the present study, a significant reduction in the amount of total phenolics was observed, albeit on a minor scale. The capacity of wheat to increase total phenolic accumulation has been directly linked to increased tolerance to numerous stressors, including drought [16,17]. On the contrary, a reduction in total phenol concentration was reported in some previous research of the tolerance of wheat and maize cultivars to osmotic stress [63,64]. Such divergent findings from research investigating the accumulation of phenolics in stressful conditions may be related to the fact that their accumulation is dependent on the plant growth stage [65]. The findings of this study indicate that the initial response of wheat shortly after the occurrence of stress conditions is a reduction of the total phenolic content in flag leaves. The response of individual phenolic acids to drought stress followed a similar trend. Significant decreases in p-coumaric, ferulic, and caffeic acid concentrations were observed in all cases, with the exception of cultivar Nada, which was able to maintain the concentrations of p-coumaric and ferulic acids consistent while increasing the concentration of caffeic acid. According to Prado et al. [66] caffeic acid showed increased synthesis under heavy metal stress in basil while the total phenolic content was least affected. When applied externally, caffeic acid helps to overcome an impact of osmotic stress in a large number of plant species, including wheat [67,68]. Additionally, Kiani et al. [16] reported a significant increase in caffeic acid content under salt stress. According to research by Guo et al. [69], caffeic acid has a significant impact on the capability of wheat seedlings to tolerate drought stress.

Chlorophyll is fundamental pigment molecule in photosynthesis playing a key role in normal growth and the development of plants. An excessive amount of ROS that appears as a result of exposure to various abiotic stressors, including drought, can affect the biosynthesis of Chl, cause degradation of thylakoid membranes, and thus, the loss of Chl content [70]. However, much research has demonstrated that an increase in Chl concentration in response to a low dose of abiotic and biotic stress enables adaptive conditioning of plants [71]. In each of the four cultivars examined in this study, the total Chl concentration increased under stress conditions. This may imply that the initial response of adult wheat to drought is an increase in Chl concentration in flag leaves in order to maintain photosynthetic activity and reduce the detrimental effects of drought stress on productivity. This was confirmed to be an early response of some other C3 plant species to heat stress [72,73]. Although the concentration of total Chl in flag leaves significantly increased, the ratio of Chl *a* to Chl *b* showed different patterns of variation. Despite the fact that the total Chl appears to be unchanged, the existence of variations in the Chl *a*/*b* ratio may indicate the degradation of Chl as a result of the exposure to different abiotic stressors [74]. In general, a higher Chl *a*/*b* ratio indicates either an increase in Chl *a* synthesis or a heightened rate of Chl *b* degradation. It has been reported that, among others, exposure to drought stress results in a greater reduction in Chl *b* concentration [75,76]. Additionally, the conversion of Chl *b* to Chl *a* occurs as part of the Chl degradation process [77]. According to Ashraf et al. [78] a greater increase in the Chl *a*/*b* ratio was observed in drought-susceptible wheat varieties compared to tolerant ones. Only cultivar Nada in the current study was able to maintain the Chl *a*/*b* ratio during drought stress, while the other three cultivars exhibited significant changes in the ratio suggesting the higher susceptibility of their photosynthetic apparatus under drought stress. Carotenoid pigments have been demonstrated as crucial ROS scavengers [11]. They are of particular importance in scavenging singlet oxygen molecules (^1^O_2_), which are produced in larger amounts as a result of stomatal closure during osmotic stress, i.e., drought and salt stress [79]. In the present study, a significant elevation in Car concentration was detected during drought stress, with the exception of the Nada cultivar, the Car concentration of which remained unaffected by drought stress.

Overall, the results obtained within this study indicate that the enzymatic antioxidant system plays the most significant role in the early response of wheat flag leaves to drought stress. Among the enzymatic antioxidant components evaluated in this study, GPOD and APX appear to play the most significant role in the early response of wheat flag leaves to drought stress and removal of an excessive H_2_O_2_. On the contrary, CAT activity seems to be less important in the early response to drought stress. However, there are some limitations in this study that should be addressed in future research. Firstly, the wheat antioxidant response to drought was estimated under artificially induced drought conditions (desiccant treatment) at the single time point after the treatment. Although being effective in gaining an understanding of the early response of field-grown wheat plants to drought, an evaluation over a longer time-period following treatment could provide a more precise assessment of the antioxidant response. Secondly, here we examined the response to drought stress of the flag leaf only due to its important role in determining the grain yield. However, the conclusions drawn from the results of flag leaf response may not necessarily reflect the response of other plant organs or provide insight into how the plant as a whole responds to drought stress.

Among the cultivars examined in this study, Nada and Dubrava revealed the strongest responses in terms of activation of the enzymatic component of the defense mechanism. Stronger activation of antioxidant enzymes, particularly GPOD and APX, in these two cultivars prevented H_2_O_2_ accumulation and, as a result, membrane damage due to lipid peroxidation. Therefore, these two cultivars can be categorized as cultivars with high drought tolerance. The remaining two cultivars (U1 and Njivka) were shown to be highly susceptible to drought stress, particularly in terms of lipid peroxidation and membrane damage. In addition to activating the enzymatic defense system, the Nada cultivar also showed an increased synthesis of the osmoprotectant proline and specific phenolic compounds such as caffeic acid, which both contribute to the increased stress tolerance. Compared to the remaining three cultivars, cultivar Nada has the broadest genetic base (Figures S1–S4 in the Supplementary Material). As shown in the Figure S1 in the Supplementary Material, various lines and cultivars originating from breeding programs of different countries were used in the development of cultivar Nada. A broader genetic base may be the reason why cultivar Nada possesses the ability to activate both enzymatic and non-enzymatic defense mechanism in an early response to drought stress. In general, the results show that biochemical and physiological responses to drought stress are genotype-dependent and are determined by the genetic base of each cultivar. Old traditional wheat cultivars with a broad genetic base can be a valuable source of drought tolerance, which is especially important given the current climate change. However, here we examined the response of a smaller number of traditional cultivars to desiccant-stimulated drought stress and a further investigation, including a higher number of traditional cultivars, is needed to make more powerful conclusions and get a better insight into the usefulness of traditional wheat cultivars in breeding for improved drought tolerance.

## 4. Materials and Methods

### 4.1. Plant Material and Experimental Conditions

Four old traditional Croatian winter wheat cultivars from the Agricultural Institute Osijek (Nada, U1, Dubrava, and Njivka) grown under field conditions were used in the present study. The selected cultivars are characterized as bread cultivars of *Triticum aestivum* L. ssp. *vulgare*. Pedigree data and the year of release of the genotypes studied are listed in Table 1, while the pedigree tree of each cultivar separately is shown in Figures S1–S4 in the Supplementary Material.

Experimental data were obtained from the field experiment conducted in the 2020/2021 growing season, at Osijek location (45°32′11.66″ N, 18°44′26.84″ E; 95 m a.s.l.). At the experimental site, the climate is predominantly continental with hot summers and cold/cool winters. The multiannual precipitation and temperature average is 674 mm and 11.2 °C, respectively, while the lowest average temperature was recorded in January (3.24 °C). The maximum average temperature of 28.1 °C occurred in July. More detailed information about meteorological data prior and during the experiment are included in Figures S5 and S6 and Table S1 in the Supplementary Material. Soil at the experimental site is characterized as eutric cambisol and detailed information on chemical properties of it is listed in Table S2 in the Supplementary Material. Sowing was performed on October 17th, 2020, with a sowing rate of 550 seeds per square meter in eight rows (between rows distance was 13.5 cm). Experimental plots were 7 m length and 1.08 m of total width, split in half on two plots of 3.78 m^2^ (treatment and control). Applied fertilizers during soil preparation (ploughing and harrowing) were 400 kg ha^−1^ of NPK 7-20-30 and 100 kg ha^−1^ of UREA 46% N. During vegetation, 120 kg ha^−1^ of KAN 27% N was applied two times, at the tillering and stem elongation growth stage. The third fertilization was applied at the beginning of heading time with 50 kg ha^−1^ of KAN 27% N, which makes a total of 153,75 kg of pure nitrogen per hectare. The suppression of diseases, weeds, and pests was carried out following the typical practice for commercial wheat production in Croatia.

The experiment was conducted at the grain filling stage (20 days after anthesis) in such a way that the flag leaves of 30 plants were randomly collected from one half of the experimental plot as control samples. All remaining plants of the other half of the plot were treated with 2% (*w*/*v*) NaClO_3_ in a water solution (pH 7.0) so that all green parts were sprayed using a 10-L hand-held pressure pump at around 500 mL per plot. The treatment was applied on the same day when control plants were collected. Desiccant-treated flag leaves from 30 plants per cultivar were collected 4 h after the treatment. Fresh leaf material (control and treated) to be used for laboratory analyses was flash-frozen in liquid nitrogen and stored at −75 °C. A fraction of that sample was freeze-dried and milled on Retsch Centrifugal Mill ZM1 (Haan, Germany) for phenolic acid analysis, and the rest of the sample was powdered in liquid nitrogen using Mill A11 (IKA, Königswinter, Germany) and used in all other biochemical analyses.

### 4.2. Water Content

A portion of the homogenized sample was weighted to obtain the fresh weight (FW) and thereafter was dried in the oven at 75 °C for 72 h to determine the dry weight (DW) [25]. The WC in the leaves was calculated according to the equation:

(1) $$\mathrm{WC}\;(\%)=\frac{\mathrm{FW}-\mathrm{DW}}{\mathrm{FW}\times 100}$$

### 4.3. Proline Content

The free proline in wheat flag leaves was extracted by ultrasound-assisted solid–liquid extraction for 60 min at 25 °C in a thermostat-controlled ultrasound bath (Sonorex RK 510H, Bandelin Electronic, Berlin, Germany). More precisely, 0.1 g of fresh powdered tissue was homogenized in 1 mL of aqueous ethanol (80% *v*/*v*) and sonicated. Supernatant was collected after centrifugation at 14,000× *g* at 4 °C for 10 min and used for the determination of free proline content [80]. Duplicate extract aliquots of 50 μL, as well as proline standards (ranged from 0.2 to 1 mM) in 80% ethanol (*v*/*v*), were mixed with 100 μL of a reaction mixture (prepared with 1% ninhydrin (*w*/*v*), 60% acetic acid (*v*/*v*), and 20% ethanol (*v*/*v*)) and kept at 95 °C for 20 min in a heating block. After cooling to the room temperature, they were spun down quickly (1 min, 500 g). An amount of 100 μL of the supernatant was transferred to a polypropylene microplate and an Epoch microplate spectrophotometer (Bio-Tek, Bad Friedrichshall, Germany) was used to read the absorbance at 520 nm. Proline content was calculated from the standard curve and expressed as μmol per g FW.

### 4.4. Lipid Peroxidation and Hydrogen Peroxide

Hydrogen peroxide content and lipid peroxidation were determined in extracts prepared from fresh powdered flag leaf tissue with 1 mL of 0.1% (*w*/*v*) trichloroacetic acid (TCA) per 0.10 g of tissue powder. Homogenates were kept in an ice bath for 15 min, and consequently centrifuged for 15 min at 14,000× *g* at 4 °C to obtain supernatants for hydrogen peroxide and lipid peroxidation assays.

#### 4.4.1. Lipid Peroxidation

Lipid peroxidation was estimated by the thiobarbituric acid (TBA) method based on production of the MDA [81]. Briefly, 0.5 mL of supernatant and 1 mL of 0.5% TBA in 20% TCA were mixed and incubated at 95 °C. After 30 min of incubation, samples were cooled in an ice bath and centrifuged at 14,000× *g* for 15 min at 4 °C. Collected supernatants were used to measure the absorbance at 532 and 600 nm in a Specord 200 spectrophotometer (Analytic Jena, Jena, Germany) against the blank (0.5% TBA in 20% TCA). The accumulated MDA was calculated using an extinction coefficient of 155 mM^−1^ cm^−1^ and expressed as nmols per gram of FW.

#### 4.4.2. Hydrogen Peroxide

The hydrogen peroxide (H_2_O_2_) concentration in extracts was quantified according to Velikova et al. [82]. The reaction started by adding 0.5 mL of 10 mM potassium phosphate buffer (pH 7.0) and 0.25 mL of 1 M potassium iodide to the 0.25 mL of sample extracts. After 20 min of dark incubation, the absorbance of the reaction mixture was read at 390 nm in an Epoch microplate spectrophotometer. H_2_O_2_ content was expressed as μmol of H_2_O_2_ per g FW based on the H_2_O_2_ calibration curve (ranged from 0.02–1 mM).

### 4.5. Antioxidative Enzymes and Polyphenol Oxidase Activity

Crude proteins were extracted from fresh powdered wheat flag leaves. Briefly, about 0.2 g of powdered leaf tissue was homogenized with 1 mL of 100 mM potassium phosphate buffer (pH 7.0) with 5 mM ascorbic acid, 0.1 mM ethylenediaminetetraacetic acid (EDTA), and 2% (*w*/*v*) polyvinylpyrrolidone (PVP). After 15 min of ice bath extraction and centrifugation for 15 min (14,000× *g*, 4 °C), re-extraction with 1 mL of the same buffer was carried out. The pooled supernatants were used for enzyme and soluble protein assays. For all enzyme assays, five extractions per sample were done, and all enzyme activities were measured in duplicate at least. The enzyme activities were expressed as nkatal per mg proteins (nkatal/mg_proteins_), which presents the catalytic transformation of 1 mol of substrates per 1 s.

#### 4.5.1. Soluble Protein Concentration

Soluble protein content was determined according to the Bradford method [83]. The reaction mixture contained 5 μL of crude protein extract, 45 μL of dH_2_O, and 1 mL of Coomassie Brilliant Blue reagent. After homogenization, reaction mixture was kept at room temperature, and after 7 min an absorbance reading at 595 nm was taken in an Epoch microplate spectrophotometer. The soluble protein content was expressed as mg per g FW based on the BSA calibration curve (ranged from 0.01–0.4 mg/mL) and used to calculate and express enzyme activities in the same crude extract.

#### 4.5.2. Catalase Activity

CAT activity was examined by measuring the initial rate of H_2_O_2_ degradation [84]. An enzymatic reaction was initialized by mixing of 10 µL of crude protein extract and 990 µL of reaction mixture (50 mM potassium phosphate buffer (pH 7.0) and 5 mM H_2_O_2_). The course of the reaction was followed by an absorbance change at 240 nm over one min.

#### 4.5.3. Ascorbate Peroxidase Activity

APX activity was traced by Nakano and Asada method [85] which records the decrease in optical density due to ASH oxidation at 290 nm over one min. The enzymatic reaction was initiated by adding 10 µL of 12 mM H_2_O_2_ in 990 µL of the reaction mixture. The reaction mixture contained 960 µL 50 mM potassium phosphate buffer (pH 7.0) with 0.1 mM EDTA, 10 µL 25 mM ascorbic acid, and 20 µL of crude protein extract.

#### 4.5.4. Guaiacol Peroxidase Activity

GPOD activity was measured by using guaiacol as a hydrogen donor according to Siegel and Galston [86]. The enzymatic reaction started by adding 5 µL of crude protein extract into 995 µL of reaction mixture (5 mM guaiacol and 2.5 mM H_2_O_2_ in 0.2 M phosphate buffer, pH 5.8). The increase in the absorbance of tetra-guaiacol at 470 nm over 1 min was used to measure GPOD activity.

#### 4.5.5. Polyphenol Oxidase Activity

PPO activity was determined as a rate of oxidation of pyrogallol to o–quinones at 40 °C [87] measured as an increase in absorbance at 430 nm over two minutes. The initiation of enzymatic reaction was started by the addition of 5 µL of crude protein extract to the reaction mixture (895 µL of 100 mM potassium phosphate buffer (pH 7.0) and 0.1 mL of 0.1 M pyrogallol).

### 4.6. Phenolic Acids Content

#### 4.6.1. Extraction of Phenolic Acids for HPLC Analysis

The extraction of phenolic acids was performed by mixing 0.1 g of lyophilized flag leaf tissue with 4 mL of acidified methanol (80% *v*/*v*; 0.1% HCl *v*/*v*). After 2 min of vortexing, the test tube with the mixture was placed in the ultrasonic bath (Sonorex RK 510H, Bandelin Electronic, Berlin, Germany) and sonicated for 10 min, following agitation in a tube rotator for 1 h at room temperature (KS 260 basic, IKA, Staufen, Germany). The mixture was then centrifuged (Universal 320R, Hettich, Tuttlingen, Germany) at 5000 rpm for 10 min. The supernatant was filtered with a nylon filter with a 0.2 μm pore size. The extract was stored at −20 °C until analysis. All extraction procedures were done in duplicate.

#### 4.6.2. HPLC Analysis of Phenolic Acids

In our study, ten phenolic acids, from which six belong to hidroxybenzoic (syringic, gallic, chlorogenic, vanillic, protocatechuic, p-hydroxybenzoic) and four to hydroxycinnamic classes (caffeic, p-coumaric, sinapic, ferulic) purchased from Sigma-Aldrich (Saint Louis, MO, USA) were analysed. Peaks were identified by comparing their relative retention times and diode array absorption spectra at 275 nm with those of the standards previously mentioned. The high-performance liquid chromatography with diode-array detection (HPLC-DAD) of our samples revealed the presence of only three hydroxycinnamic acids (caffeic, p-coumaric and ferulic) which were further quantified using a five-point external calibration curve (R^2^ ≥ 0.999). The total phenolic content represented a “sum of phenolics” and was expressed as ferulic acid equivalents from the total area of all peaks under chromatogram, including the unidentified ones. The phenolics composition analysis of each extract was carried out in duplicate.

### 4.7. Photosynthetic Pigments

Photosynthetic pigments were extracted from fresh powdered flag leaf tissue with absolute acetone. Concentrations of total Chl (Chl *a*+*b*), carotenoids (Car), Chl *a*/*b* ratio, and Chl *a*+*b*/Car ratio were calculated from absorbance readings at 470 nm, 647 nm, and 663 nm [88] by a Specord 200 spectrophotometer (Analytik, Jena, Germany). Photosynthetic pigment concentrations were expressed as mg per g FW.

### 4.8. Data Analysis

The analysis of variance was carried out to determine differences among the treatments and varieties. Mean comparisons were done using the least significant difference (LSD) test at the 0.05 level of probability. Statistical analysis was performed within the R environment [89]. Results in all figures are presented as means ± standard error of five replicates.

## Figures and Tables

**Table 1:** Name, pedigree data, and year of release of genotypes used in the present study.

Name	Pedigree	Year of Release
Nada	OSJECKA-20/OSK-4.216-2-76	1984
U1	CARLOTTA STRAMPELLI/MARQUIS	1936
Dubrava	U1/PILOT//LIBERO	1968
Njivka	SLAVONKA/OSK-5-132-2-74	1987

## Data Availability

The data were obtained from the Agricultural Institute Osijek and are available on request from the corresponding author with the permission of the Agricultural Institute Osijek.

## Supplementary Materials

The following supporting information can be downloaded at: https://www.mdpi.com/article/10.3390/plants12020249/s1, Figure S1: Pedigree tree of cultivar Nada; Figure S2: Pedigree tree of cultivar U1; Figure S3: Pedigree tree of cultivar Dubrava; Figure S4: Pedigree tree of cultivar Njivka; Figure S5: Average air temperatures (°C), leaf temperatures (°C), and soil temperatures at 5 cm soil depth (°C) in the period before and after the experiment was conducted. Vertical red line indicates the exact date and conditions present at the time of the experiment; Figure S6: Average rainfall (mm), air moisture (%), and soil moisture (cbar) in the period before and after the experiment was conducted. Vertical red line indicates the exact date and conditions present at the time of the experiment; Table S1: The weather and soil condition data at the beginning of the experiment collected from the field meteorological observatory located at the experimental site (Agricultural Institute Osijek); Table S2: The chemical properties of soil at the experimental site (Agricultural Institute Osijek).

## References

[1] Raza A., Razzaq A., Mehmood S.S., Zou X., Zhang X., Lv Y., Xu J. (2019). Impact of Climate Change on Crops Adaptation and Strategies to Tackle Its Outcome: A Review. Plants.

[2] Shewry P.R., Hey S.J. (2015). The Contribution of Wheat to Human Diet and Health. Food Energy Secur..

[3] Arora N.K. (2019). Impact of Climate Change on Agriculture Production and Its Sustainable Solutions. Environ. Sustain..

[4] Marinović I., Kalin K.C., Güttler I., Pasarić Z. (2021). Dry Spells in Croatia: Observed Climate Change and Climate Projections. Atmosphere.

[5] Barić M., Kereša S., Jerčić I.H., Havrda S., Gelenčir D. (2008). Evaluation and Characterization of Croatian Winter Wheat Genotypes (*T. aestivum* L.) for Drought Tolerance. Cereal Res. Commun..

[6] Laxa M., Liebthal M., Telman W., Chibani K., Dietz K.J. (2019). The Role of the Plant Antioxidant System in Drought Tolerance. Antioxidants.

[7] De Carvalho M.H.C. (2008). Drought Stress and Reactive Oxygen Species: Production, Scavenging and Signaling. Plant Signal. Behav..

[8] Blum A. (2011). Drought Resistance and Its Improvement. Plant Breeding for Water-Limited Environments.

[9] Mwadzingeni L., Figlan S., Shimelis H., Mondal S., Tsilo T.J. (2017). Genetic Resources and Breeding Methodologies for Improving Drought Tolerance in Wheat. J. Crop Improv..

[10] Mittler R., Vanderauwera S., Suzuki N., Miller G., Tognetti V.B., Vandepoele K., Gollery M., Shulaev V., Van Breusegem F. (2011). ROS Signaling: The New Wave?. Trends Plant Sci..

[11] Gill S.S., Tuteja N. (2010). Reactive Oxygen Species and Antioxidant Machinery in Abiotic Stress Tolerance in Crop Plants. Plant Physiol. Biochem..

[12] Alché J.D.D. (2019). A Concise Appraisal of Lipid Oxidation and Lipoxidation in Higher Plants. Redox Biol..

[13] Rawat N., Singla-Pareek S.L., Pareek A. (2021). Membrane Dynamics during Individual and Combined Abiotic Stresses in Plants and Tools to Study the Same. Physiol. Plant..

[14] Simova-Stoilova L., Vaseva I., Grigorova B., Demirevska K., Feller U. (2010). Proteolytic Activity and Cysteine Protease Expression in Wheat Leaves under Severe Soil Drought and Recovery. Plant Physiol. Biochem..

[15] Upadhyay D., Budhlakoti N., Singh A.K., Bansal R., Kumari J., Chaudhary N., Padaria J.C., Sareen S., Kumar S. (2020). Drought Tolerance in *Triticum aestivum* L. Genotypes Associated with Enhanced Antioxidative Protection and Declined Lipid Peroxidation. 3 Biotech.

[16] Kiani R., Arzani A., Mirmohammady Maibody S.A.M. (2021). Polyphenols, Flavonoids, and Antioxidant Activity Involved in Salt Tolerance in Wheat, Aegilops Cylindrica and Their Amphidiploids. Front. Plant Sci..

[17] Naderi S., Fakheri B.A., Maali-Amiri R., Mahdinezhad N. (2020). Tolerance Responses in Wheat Landrace Bolani Are Related to Enhanced Metabolic Adjustments under Drought Stress. Plant Physiol. Biochem..

[18] Dumanović J., Nepovimova E., Natić M., Kuča K., Jaćević V. (2021). The Significance of Reactive Oxygen Species and Antioxidant Defense System in Plants: A Concise Overview. Front. Plant Sci..

[19] Andrés C.M.C., de la Lastra J.M.P., Juan C.A., Plou F.J., Pérez-Lebeña E. (2022). Chemistry of Hydrogen Peroxide Formation and Elimination in Mammalian Cells, and Its Role in Various Pathologies. Stresses.

[20] Quan L.J., Zhang B., Shi W.W., Li H.Y. (2008). Hydrogen Peroxide in Plants: A Versatile Molecule of the Reactive Oxygen Species Network. J. Integr. Plant Biol..

[21] Herbette S., Lenne C., Leblanc N., Julien J.L., Drevet J.R., Roeckel-Drevet P. (2002). Two GPX-like Proteins from Lycopersicon Esculentum and Helianthus Annuus Are Antioxidant Enzymes with Phospholipid Hydroperoxide Glutathione Peroxidase and Thioredoxin Peroxidase Activities. Eur. J. Biochem..

[22] Sharma P., Jha A.B., Dubey R.S., Pessarakli M. (2012). Reactive Oxygen Species, Oxidative Damage, and Antioxidative Defense Mechanism in Plants under Stressful Conditions. J. Bot..

[23] Asada K. (1999). The Water-Water Cycle in Chloroplasts: Scavenging of Active Oxygens and Dissipation of Excess Photons. Annu. Rev. Plant Biol..

[24] Hernández I., Cela J., Alegre L., Munné-Bosch S., Aroca R. (2012). Antioxidant Defenses against Drought Stress. Plant Responses to Drought Stress.

[25] Kumar D., Al Hassan M., Naranjo M.A., Agrawal V., Boscaiu M., Vicente O. (2017). Effects of Salinity and Drought on Growth, Ionic Relations, Compatible Solutes and Activation of Antioxidant Systems in Oleander (*Nerium oleander* L.). PLoS ONE.

[26] Chakraborty U., Pradhan B. (2012). Drought Stress-Induced Oxidative Stress and Antioxidative Responses in Four Wheat (*Triticum aestivum* L.) Varieties. Arch. Agron. Soil Sci..

[27] Ma D., Sun D., Wang C., Li Y., Guo T. (2014). Expression of Flavonoid Biosynthesis Genes and Accumulation of Flavonoid in Wheat Leaves in Response to Drought Stress. Plant Physiol. Biochem..

[28] Boeckx T., Winters A.L., Webb K.J., Kingston-Smith A.H. (2015). Polyphenol Oxidase in Leaves: Is There Any Significance to the Chloroplastic Localization?. J. Exp. Bot..

[29] Sofo A., Dichio B., Xiloyannis C., Masia A. (2005). Antioxidant Defences in Olive Trees during Drought Stress: Changes in Activity of Some Antioxidant Enzymes. Funct. Plant Biol..

[30] Thipyapong P., Melkonian J., Wolfe D.W., Steffens J.C. (2004). Suppression of Polyphenol Oxidases Increases Stress Tolerance in Tomato. Plant Sci..

[31] Lee B.R., Kim K.Y., Jung W.J., Avice J.C., Ourry A., Kim T.H. (2007). Peroxidases and Lignification in Relation to the Intensity of Water-Deficit Stress in White Clover (*Trifolium repens* L.). J. Exp. Bot..

[32] Blum A. (2017). Osmotic Adjustment Is a Prime Drought Stress Adaptive Engine in Support of Plant Production. Plant Cell Environ..

[33] Ashraf M., Foolad M.R. (2007). Roles of Glycine Betaine and Proline in Improving Plant Abiotic Stress Resistance. Environ. Exp. Bot..

[34] Trovato M., Mattioli R., Costantino P. (2008). Multiple Roles of Proline in Plant Stress Tolerance and Development. Rend. Lincei.

[35] Mwadzingeni L., Shimelis H., Tesfay S., Tsilo T.J. (2016). Screening of Bread Wheat Genotypes for Drought Tolerance Using Phenotypic and Proline Analyses. Front. Plant Sci..

[36] Blum A., Tuberosa R. (2018). Dehydration Survival of Crop Plants and Its Measurement. J. Exp. Bot..

[37] Dodig D., Zorić M., Jović M., Kandić V., Stanisavljević R., Šurlan-Momirović G. (2015). Wheat Seedlings Growth Response to Water Deficiency and How It Correlates with Adult Plant Tolerance to Drought. J. Agric. Sci..

[38] Breinl K., Di Baldassarre G., Mazzoleni M., Lun D., Vico G. (2020). Extreme Dry and Wet Spells Face Changes in Their Duration and Timing. Environ. Res. Lett..

[39] Zhang C.J., Chen G.X., Gao X.X., Chu C.J. (2006). Photosynthetic Decline in Flag Leaves of Two Field-Grown Spring Wheat Cultivars with Different Senescence Properties. S. Afr. J. Bot..

[40] Fischer R.A., Maurer R. (1978). Drought Resistance in Spring Wheat Cultivars. I. Grain Yield Responses. Aust. J. Agric. Res..

[41] Ozturk A., Aydin F. (2004). Effect of Water Stress at Various Growth Stages on Some Quality Characteristics of Winter Wheat. J. Agron. Crop Sci..

[42] Allahverdiyev T.I. (2015). Effect of Drought Stress on Some Physiological Traits of Durum (*Triticum durum* Desf.) and Bread (*Triticum aestivum* L.) Wheat Genotypes. J. Stress Physiol. Biochem..

[43] Schonfeld M.A., Johnson R.C., Carver B.F., Mornhinweg D.W. (1988). Water Relations in Winter Wheat as Drought Resistance Indicators. Crop Sci..

[44] Chowdhury M.K., Hasan M.A., Bahadur M.M., Islam M.R., Hakim M.A., Iqbal M.A., Javed T., Raza A., Shabbir R., Sorour S. (2021). Evaluation of Drought Tolerance of Some Wheat (*Triticum aestivum* L.) Genotypes through Phenology, Growth, and Physiological Indices. Agronomy.

[45] Baloch M.J., Dunwell J., Khan N.U., Jatoi W.A., Khakhwani A.A., Vessar N.F., Gul S. (2013). Morpho-Physiological Characterization of Spring Wheat Genotypes under Drought Stress. Int. J. Agric. Biol..

[46] Larbi A., Mekliche A., Agronomique I.N., De Phytotechnie D. (2004). Relative Water Content (RWC) and Leaf Senescence as Screening Tools for Drought Tolerance in Wheat. Growth.

[47] Maghsoudi K., Emam Y., Niazi A., Pessarakli M., Arvin M.J. (2018). P5CS Expression Level and Proline Accumulation in the Sensitive and Tolerant Wheat Cultivars under Control and Drought Stress Conditions in the Presence/Absence of Silicon and Salicylic Acid. J. Plant Interact..

[48] Bowne J.B., Erwin T.A., Juttner J., Schnurbusch T., Langridge P., Bacic A., Roessner U. (2012). Drought Responses of Leaf Tissues from Wheat Cultivars of Differing Drought Tolerance at the Metabolite Level. Mol. Plant.

[49] Sultan M.A.R.F., Hui L., Yang L.J., Xian Z.H. (2012). Assessment of Drought Tolerance of Some *Triticum* L. Species through Physiological Indices. Czech J. Genet. Plant Breed..

[50] Ahmed I.M., Dai H., Zheng W., Cao F., Zhang G., Sun D., Wu F. (2013). Genotypic Differences in Physiological Characteristics in the Tolerance to Drought and Salinity Combined Stress between Tibetan Wild and Cultivated Barley. Plant Physiol. Biochem..

[51] Sachdev S., Ansari S.A., Ansari M.I., Fujita M., Hasanuzzaman M. (2021). Abiotic Stress and Reactive Oxygen Species: Generation, Signaling, and Defense Mechanisms. Antioxidants.

[52] Abid M., Ali S., Qi L.K., Zahoor R., Tian Z., Jiang D., Snider J.L., Dai T. (2018). Physiological and Biochemical Changes during Drought and Recovery Periods at Tillering and Jointing Stages in Wheat (*Triticum aestivum* L.). Sci. Rep..

[53] Hameed A., Goher M., Iqbal N. (2013). Drought Induced Programmed Cell Death and Associated Changes in Antioxidants, Proteases, and Lipid Peroxidation in Wheat Leaves. Biol. Plant..

[54] Peršić V., Ament A., Dunić J.A., Drezner G., Cesar V. (2022). PEG-Induced Physiological Drought for Screening Winter Wheat Genotypes Sensitivity—Integrated Biochemical and Chlorophyll a Fl Uorescence Analysis. Front. Plant Sci..

[55] Mittler R. (2002). Oxidative Stress, Antioxidants and Stress Tolerance. Trends Plant Sci..

[56] Arora A., Sairam R.K., Srivastava G.C. (2002). Oxidative Stress and Antioxidative System in Plants. Curr. Sci..

[57] Vuković R., Čamagajevac I.Š., Vuković A., Šunić K., Begović L., Mlinarić S., Sekulić R., Sabo N., Španić V. (2022). Physiological, Biochemical and Molecular Response of Different Winter Wheat Varieties under Drought Stress at Germination and Seedling Growth Stage. Antioxidants.

[58] Pour-Benab S.M., Fabriki-Ourang S., Mehrabi A.A. (2019). Expression of Dehydrin and Antioxidant Genes and Enzymatic Antioxidant Defense under Drought Stress in Wild Relatives of Wheat. Biotechnol. Biotechnol. Equip..

[59] Sullivan M.L. (2015). Beyond Brown: Polyphenol Oxidases as Enzymes of Plant Specialized Metabolism. Front. Plant Sci..

[60] Gandía-Herrero F., García-Carmona F. (2013). Biosynthesis of Betalains: Yellow and Violet Plant Pigments. Trends Plant Sci..

[61] Boeckx T., Webster R., Winters A.L., Webb K.J., Gay A., Kingston-Smith A.H. (2015). Polyphenol Oxidase-Mediated Protection against Oxidative Stress Is Not Associated with Enhanced Photosynthetic Efficiency. Ann. Bot..

[62] Boeckx T., Winters A., Webb K.J., Kingston-Smith A.H. (2017). Detection of Potential Chloroplastic Substrates for Polyphenol Oxidase Suggests a Role in Undamaged Leaves. Front. Plant Sci..

[63] Rao A., Ahmad S.D., Sabir S.M., Awan S.I., Hameed A., Abbas S.R., Shehzad M., Khan M.F., Shafique S., Ahmad Z. (2013). Detection of Saline Tolerant Wheat Cultivars (*Triticum aestivum* L.) Using Lipid Peroxidation, Antioxidant Defense System, Glycine-Betaine and Proline Contents. J. Anim. Plant Sci..

[64] Moharramnejad S., Sofalian O., Valizadeh M., Asgari A., Shiri M. (2015). Proline, Glycine Betaine, Total Phenolics and Pigment Contents in Response to Osmotic Stress in Maize Seedlings. J. Biosci. Biotechnol..

[65] Ashraf M.A., Ashraf M., Ali Q. (2010). Response of Two Genetically Diverse Wheat Cultivars to Salt Stress at Different Growth Stages: Leaf Lipid Peroxidation and Phenolic Contents. Pak. J. Bot..

[66] Prado N.B.D., de Abreu C.B., Pinho C.S., Junior M.M.d.N., Silva M.D., Espino M., Silva M.F., Dias F.D.S. (2022). Application of Multivariate Analysis to Assess Stress by Cd, Pb and Al in Basil (*Ocimum basilicum* L.) Using Caffeic Acid, Rosmarinic Acid, Total Phenolics, Total Flavonoids and Total Dry Mass in Response. Food Chem..

[67] Zafar-Ul-Hye M., Akbar M.N., Iftikhar Y., Abbas M., Zahid A., Fahad S., Datta R., Ali M., Elgorban A.M., Ansari M.J. (2021). Rhizobacteria Inoculation and Caffeic Acid Alleviated Drought Stress in Lentil Plants. Sustainability.

[68] Mehmood H., Abbasi G.H., Jamil M., Malik Z., Ali M., Iqbal R. (2021). Assessing the Potential of Exogenous Caffeic Acid Application in Boosting Wheat (*Triticum aestivum* L.) Crop Productivity under Salt Stress. PLoS ONE.

[69] Guo X., Xin Z., Yang T., Ma X., Zhang Y., Wang Z., Ren Y., Lin T. (2020). Metabolomics Response for Drought Stress Tolerance. Plants.

[70] Sharma A., Kumar V., Shahzad B., Ramakrishnan M., Singh Sidhu G.P., Bali A.S., Handa N., Kapoor D., Yadav P., Khanna K. (2020). Photosynthetic Response of Plants Under Different Abiotic Stresses: A Review. J. Plant Growth Regul..

[71] Agathokleous E., Feng Z.Z., Peñuelas J. (2020). Chlorophyll Hormesis: Are Chlorophylls Major Components of Stress Biology in Higher Plants?. Sci. Total Environ..

[72] Vuletić M.V., Mihaljević I., Tomaš V., Horvat D., Zdunić Z., Vuković D. (2022). Physiological Response to Short-Term Heat Stress in the Leaves of Traditional and Modern Plum (*Prunus domestica* L.) Cultivars. Horticulturae.

[73] Gür A., Demirel U., Özden M., Kahraman A., Çopur O. (2010). Diurnal Gradual Heat Stress Affects Antioxidant Enzymes, Proline Accumulation and Some Physiological Components in Cotton (*Gossypium hirsutum* L.). Afr. J. Biotechnol..

[74] Ashraf M., Harris P.J.C. (2013). Photosynthesis under Stressful Environments: An Overview. Photosynthetica.

[75] Jaleel C.A., Manivannan P., Wahid A., Farooq M., Al-Juburi H.J., Somasundaram R., Panneerselvam R. (2009). Drought Stress in Plants: A Review on Morphological Characteristics and Pigments Composition. Int. J. Agric. Biol..

[76] Jain M., Tiwary S., Gadre R. (2010). Sorbitol-Induced Changes in Various Growth and Biochemical Parameters in Maize. Plant Soil Environ..

[77] Hu X., Gu T., Khan I., Zada A. (2021). Research Progress in the Interconversion, Turnover and Degradation of Chlorophyll. Cells.

[78] Ashraf M.Y., Azmi A.R., Khan A.H., Ala S. (1994). Effect of Water Stress on Total Phenol, Peroxidase Activity and Chlorophyll Contents in Wheat (*Triticum aestivum* L.). Acta Physiol. Plant..

[79] Krieger-Liszkay A., Fufezan C., Trebst A. (2008). Singlet Oxygen Production in Photosystem II and Related Protection Mechanism. Photosynth. Res..

[80] Woodrow P., Ciarmiello L.F., Annunziata M.G., Pacifico S., Iannuzzi F., Mirto A., D’Amelia L., Dell’Aversana E., Piccolella S., Fuggi A. (2017). Durum Wheat Seedling Responses to Simultaneous High Light and Salinity Involve a Fine Reconfiguration of Amino Acids and Carbohydrate Metabolism. Physiol. Plant..

[81] Verma S., Dubey R.S. (2003). Lead Toxicity Induces Lipid Peroxidation and Alters the Activities of Antioxidant Enzymes in Growing Rice Plants. Plant Sci..

[82] Velikova V., Yordanov I., Edreva A. (2000). Oxidative Stress and Some Antioxidant Systems in Acid Rain-Treated Bean Plants: Protective Role of Exogenous Polyamines. Plant Sci..

[83] Bradford M.M. (1976). A Rapid and Sensitive Method for the Quantitation of Microgram Quantitites of Protein Utilizing the Principle of Protein-Dye Binding. Anal. Biochem..

[84] Aebi H. (1984). Catalase In Vitro. Methods in Enzymology.

[85] Nakano Y., Asada K. (1981). Hydrogen Peroxide Is Scavenged by Ascorbate-Specific Peroxidase in Spinach Chloroplasts. Plant Cell Physiol..

[86] Siegel B.Z., Galston W. (1967). The Isoperoxidases of *Pisum sativum*. Plant Physiol..

[87] Raymond J., Rakariyatham N., Azanza J.L. (1993). Purification and Some Properties of Polyphenoloxidase from Sunflower Seeds. Phytochemistry.

[88] Lichtenthaler H.K. (1987). Chlorophylls and Carotenoids: Pigments of Photosynthetic Biomembranes. Methods Enzymol..

[89] R Core Team (2022). R: A Language and Environment for Statistical Computing.

