# Supplementary material for: Early Antioxidative Response to Desiccant-Stimulated Drought Stress in Field-Grown Traditional Wheat Varieties
Source: Plants (Basel). 2023 Jan 5;12(2):249. doi: 10.3390/plants12020249 (PMC9867156; doi:10.3390/plants12020249)
Supplement: Supplementary file 1 [file plants-12-00249-s001.zip › plants-2069679-supplementary.pdf]

Supplementary Material

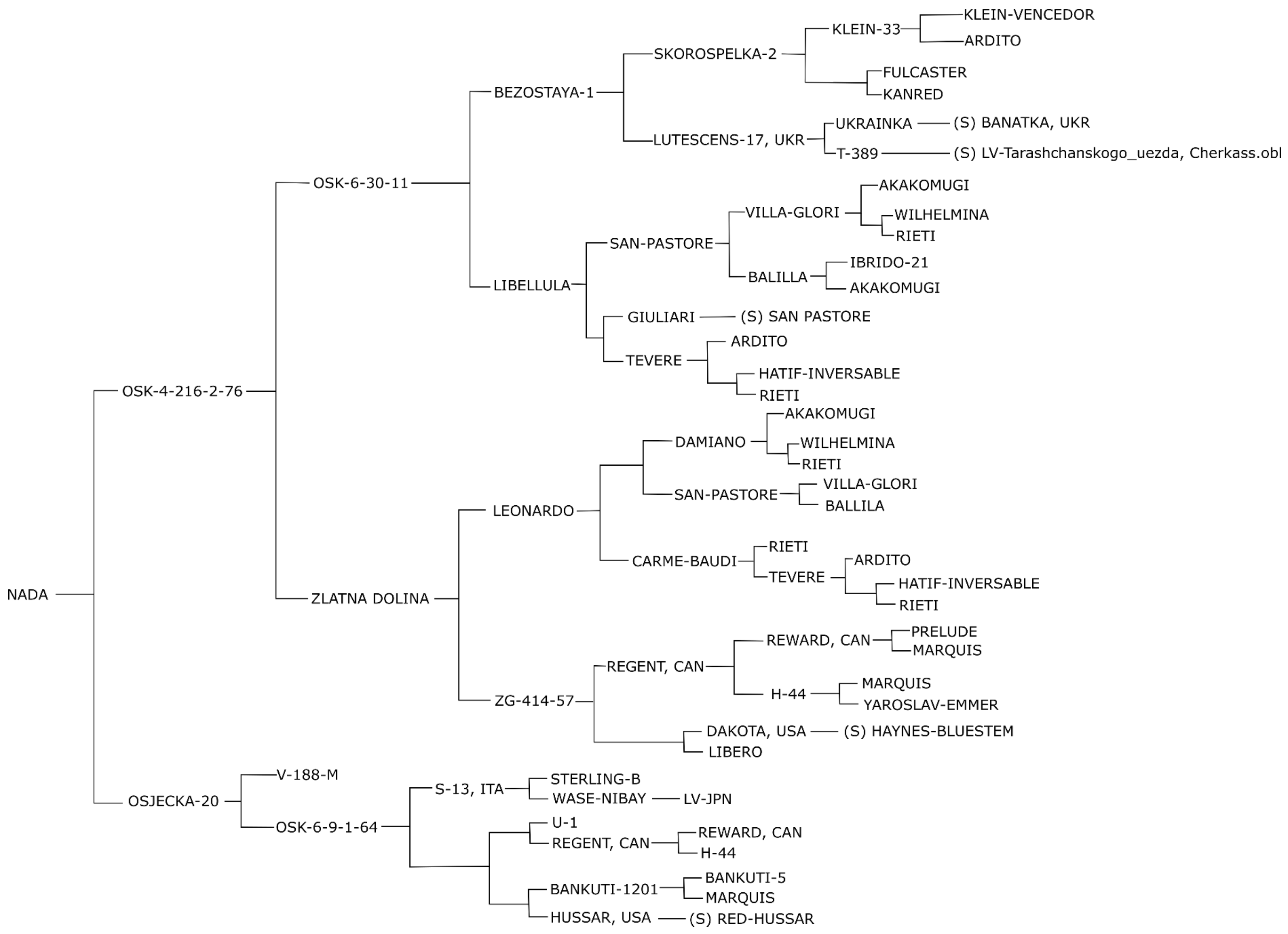

Figure S1. Pedigree tree of cultivar Nada.

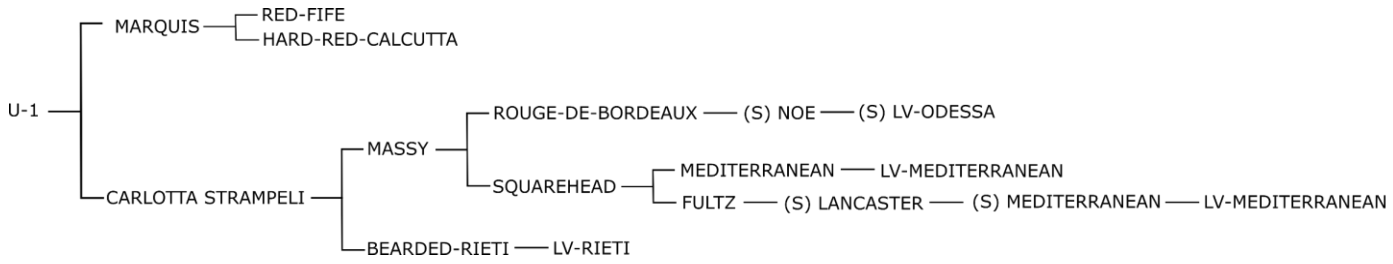

Figure S2. Pedigree tree of cultivar U1.

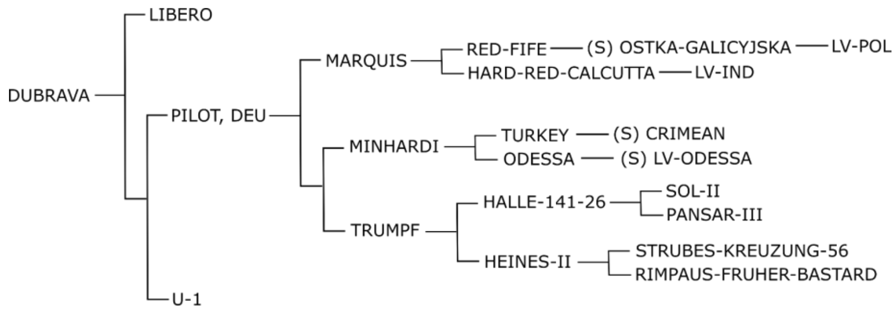

Figure S3. Pedigree tree of cultivar Dubrava.

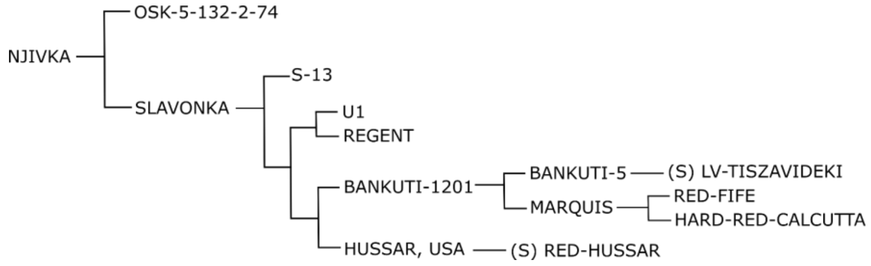

Figure S4. Pedigree tree of cultivar Njivka.

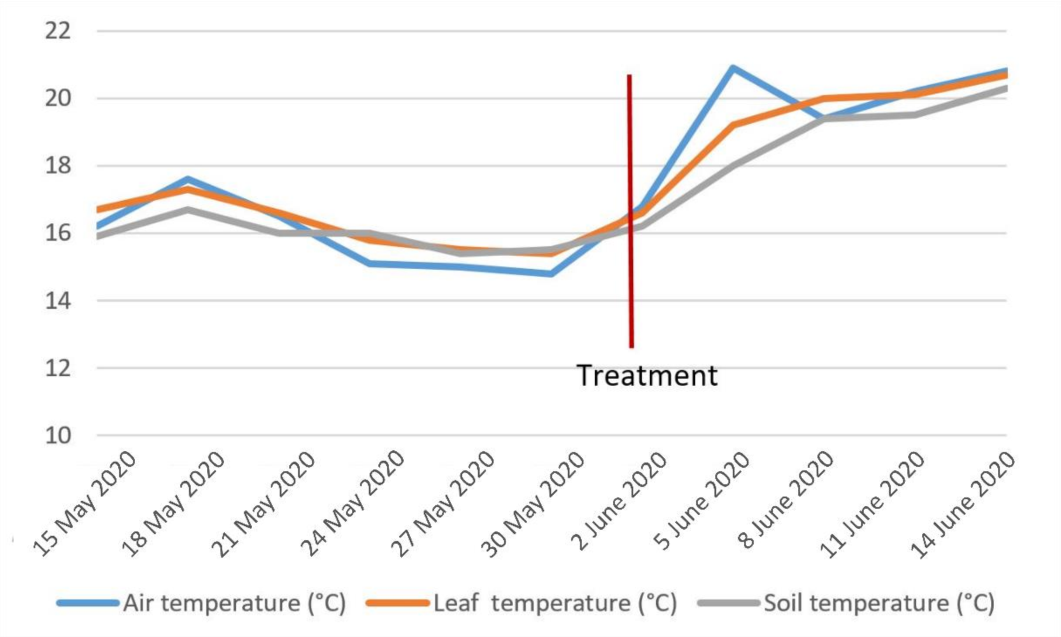

Figure S5. Average air temperatures (°C), leaf temperatures (°C), and soil temperatures at 5 cm soil depth (°C) in the period before and after the experiment was conducted. Vertical red line indicates the exact date and conditions present at the time of the experiment. The data are collected from the field meteorological observatory located at the experimental site (Agricultural Institute Osijek).

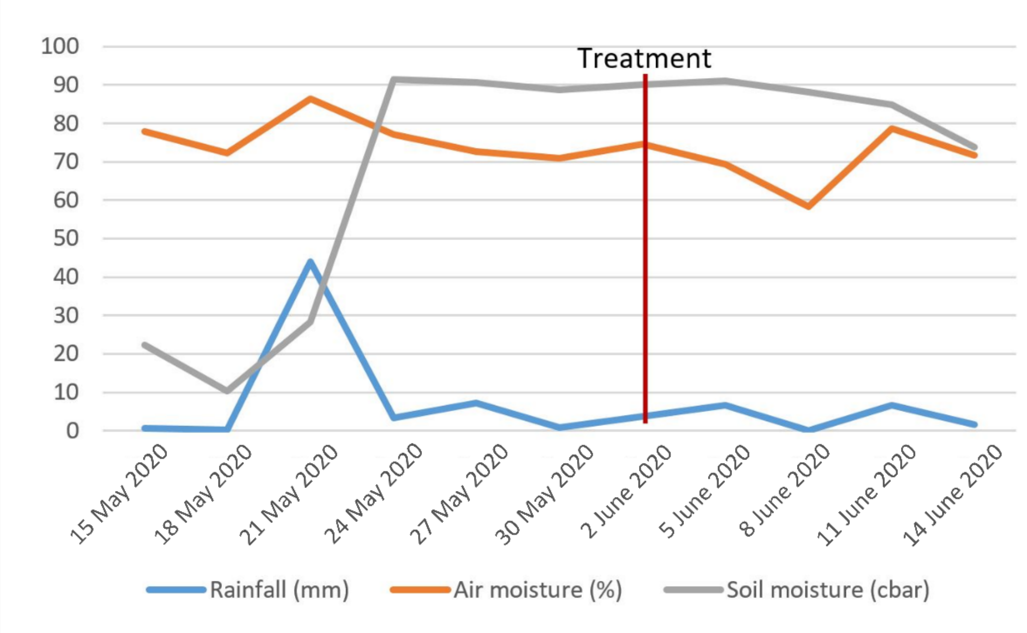

Figure S6. Average rainfall (mm), air moisture (%), and soil moisture (cbar) in the period before and after the experiment was conducted. Vertical red line indicates the exact date and conditions present at the time of the experiment. The data are collected from the field meteorological observatory located at the experimental site (Agricultural Institute Osijek).

Table S1. The weather and soil condition data at the beginning of the experiment collected from the field meteorological observatory located at the experimental site (Agricultural Institute Osijek).

Date and time	Air temperature (°C)	Leaf temperature (°C)	Soil temperature (°C)	Air moisture (%)	Soil moisture (cbar)	Rain (mm)
2 June 2020 9:00:00	16.7	15.5	15.3	54.6	87.6	0

Table S2. The chemical properties of soil at the experimental site (Agricultural Institute Osijek).

Soil type	pH		Humus (%)	N (%)	P_2O_5	K_2O
	H_2O	KCl			mg/100 g	mg/100 g
Eutric cambisol	6.67	7.12	1.8	0.11	22.59	35.01
